# Supplementary material for: Role and mechanism of NCAPD3 in promoting malignant behaviors in gastric cancer
Source: Front Pharmacol. 2024 Apr 22;15:1341039. doi: 10.3389/fphar.2024.1341039 (PMC11070777; doi:10.3389/fphar.2024.1341039)
Supplement: Supplementary file 11 [file DataSheet2.ZIP › GSEA/GO_CC/my_analysis.Gsea.1599461408869/GO_TIGHT_JUNCTION.html]

Details for gene set GO\_TIGHT\_JUNCTION[GSEA]

|  || Dataset | filtered\_dataset.sample\_info.cls#WT\_versus\_NCAPD3\_MUT |
| Phenotype | sample\_info.cls#WT\_versus\_NCAPD3\_MUT |
| Upregulated in class | WT |
| GeneSet | GO\_TIGHT\_JUNCTION |
| Enrichment Score (ES) | 0.28445125 |
| Normalized Enrichment Score (NES) | 1.1145892 |
| Nominal p-value | 0.3246528 |
| FDR q-value | 0.8860362 |
| FWER p-Value | 1.0 |
Table: GSEA Results Summary

  

Fig 1: Enrichment plot: GO\_TIGHT\_JUNCTION      
 Profile of the Running ES Score & Positions of GeneSet Members on the Rank Ordered List

  

| SYMBOL | TITLE | RANK IN GENE LIST | RANK METRIC SCORE | RUNNING ES | CORE ENRICHMENT || 1 | 23322 | RPGRIP1L | 60 | 0.871 | 0.0499 | Yes |
| 2 | 10207 | INADL | 73 | 0.851 | 0.1320 | Yes |
| 3 | 92140 | MTDH | 93 | 0.801 | 0.2038 | Yes |
| 4 | 56288 | PARD3 | 99 | 0.790 | 0.2845 | Yes |
| 5 | 324 | APC | 281 | 0.607 | 0.2194 | No |
| 6 | 9075 | CLDN2 | 420 | 0.522 | 0.1761 | No |
| 7 | 55870 | ASH1L | 436 | 0.512 | 0.2199 | No |
| 8 | 23586 | DDX58 | 584 | 0.433 | 0.1607 | No |
| 9 | 153562 | MARVELD2 | 732 | 0.359 | 0.0935 | No |
| 10 | 9223 | MAGI1 | 737 | 0.357 | 0.1287 | No |
| 11 | 54532 | USP53 | 898 | -0.322 | 0.0482 | No |
| 12 | 9181 | ARHGEF2 | 902 | -0.326 | 0.0808 | No |
| 13 | 9414 | TJP2 | 916 | -0.345 | 0.1083 | No |
| 14 | 9076 | CLDN1 | 1084 | -0.463 | 0.0379 | No |
| 15 | 90952 | ESAM | 1149 | -0.514 | 0.0468 | No |
| 16 | 4301 | MLLT4 | 1237 | -0.614 | 0.0499 | No |
| 17 | 55691 | FRMD4A | 1295 | -0.689 | 0.0825 | No |
Table: GSEA details [plain text format]

  

Fig 2: GO\_TIGHT\_JUNCTION      
 Blue-Pink O' Gram in the Space of the Analyzed GeneSet

  

Fig 3: GO\_TIGHT\_JUNCTION: Random ES distribution      
 Gene set null distribution of ES for **GO\_TIGHT\_JUNCTION**
